# Using seasonal physiological and biochemical responses to select forest components adapted to soybean and corn intercropping

**DOI:** 10.1016/j.heliyon.2024.e34674

**Published:** 2024-07-24

**Authors:** Érica Letícia Gomes Costa, Thales Caetano de Oliveira, Alex Rodrigues Gomes, Carlos Henrique Pereira Bento, Fabia Barbosa da Silva, Estenio Moreira Alves, Tiago do Prado Paim, Fabiano Guimarães Silva

**Affiliations:** a Instituto Federal de Educação, Ciência e Tecnologia Goiano, Rio Verde Campus, Rodovia Sul Goiana, Km 01, Zona Rural, Rio Verde, 75.901-970, GO, Brazil; b Rede Pró-Centro Oeste, Instituto Federal de Educação, Ciência e Tecnologia Goiano, Rio Verde Campus, Rodovia Sul Goiana, Km 01, Zona Rural, Rio Verde, 75.901-970, GO, Brazil; c Instituto Federal de Educação, Ciência e Tecnologia Goiano, Iporá Campus, Avenida Oeste, 350, Parque União, Iporá, 76200-000, GO, Brazil

**Keywords:** Defense mechanisms, Drought tolerance, *Eucalyptus* genotypes, Native trees, Plant physiology, Water use efficiency

## Abstract

Given the increasing utilization of forest components in integration systems worldwide, coupled with the growing demand for food in regions facing water restrictions, this study aims to evaluate how physiological and biochemical parameters contribute to the diversification of adaptive mechanisms among native species and eucalyptus genotypes intercropped with soybean or corn. The native tree species *Anadenanthera macrocarpa* and *Dipteryx alata*, and the eucalyptus genotypes Urograndis I-144 and Urocam VM01, were grown in soybean and corn intercropping areas and evaluated in fall, winter, spring, and summer. The study evaluated morning water potential, chloroplast pigment concentration, gas exchange, cell damage, and antioxidant enzyme activity. Intercropped with soybean, development the of *A. macrocarpa* improved through instantaneous water use efficiency, energy use by the electron transport chain, chloroplast pigments, and catalase enzyme activity. On the other hand, *A. macrocarpa* when, intercropped with corn, despite increasing energy absorption by the reaction center, there is a need for non-photochemical dissipation and in the activity of the enzymes superoxide dismutase and ascorbate peroxidase in response to water and oxidative deficits. In *D. alata*, the physiological and biochemical responses were not influenced by intercropping but by seasons, with increased chloroplast pigments in fall and electron transport in summer. However, in corn intercropping, the dissipation of excess energy allowed leaf acclimatization. The I-144 and VM01 genotypes also showed no significant differences between intercrops. The results describe photosynthetic and biochemical challenges in the native species *A. macrocarpa* intercropped with corn, such as a greater need for enzymatic and non-enzymatic defense mechanisms in response to more negative water potential. In *D. alata*, the challenges are present in both intercrops due to improved mechanisms to protect the photosynthetic apparatus. The survival of the I-144 genotype may be inefficient in both intercrops under prolonged drought conditions, as it modifies the photosystem; in contrast, genotype VM01 was the most adapted to the system for using captured energy, reducing water loss and being resilient.

## 1 Introduction

Integrated systems are strategic sustainable, agricultural production systems recognized as an excellent strategy for intensifying production [1]. These systems follow the principles of sustainable conservation agriculture proposed by the United Nations for 2030 [2]. Maximizing the synergy between components optimizes land use, diversifies production, and improves soil quality. The introduction of plant/tree species helps build more sustainable agriculture [[3], [4], [5], [6]].

Trees properly managed in integration systems can change microclimatic conditions in the growing environment, reducing photosynthetically active radiation (PAR) and wind speed [7,8]. In turn, they affect evapotranspiration, soil moisture content, and the system's productivity. Thus, decision-making on forest species should prioritize factors such as soil and water conservation [9] to reduce climate change effects on agricultural systems [10]. As climate change intensifies, the physiological performance of the forest component tends to decrease, making it important to select tree species that are adaptable to a broad range of climatic conditions, as they would be more adaptable to climate change effects in the long-term [11]. However, little is known about the physiological behavior of forest species in these systems.

The response of the forest component in the system can vary according to the species, age of the tree, geographical location, and climatic factors [12]. Choosing the right forest species for an integration system is one of the most important steps for ensuring its success [13]. Traditionally, the most cultivated forest species are exotic species [14], with the predominantly Australian genus *Eucalyptus* widely used in Brazil. However, native species such as *Dipteryx alata*, which produces fine wood and fruit with a good market value, and *Anadenanthera macrocarpa*, which also produces superb timber [15], need to be better studied. Therefore, a physiological and biochemical understanding of these species when intercropped with annual crops such as soybean and corn would complement existing practices, avoid additional costs, and improve the system's diversity and economic gain.

The need to understand the complexity of the forest component in the integration system has been recognized over the last decade, both in terms of physiological responses and adaptations to intercropping systems with annual crops. Forest component intercrops with soybean and corn can increase the performance of the forest component system, but these results may be unpredictable depending on the cultivation array. The forest component can use several adaptation strategies that may indicate periods when the intercropping system can compete with the forest component and conditions of low water availability [16]. Adaptation strategies can be observed in trees specifically through improved water use efficiency to maximize carbon storage, conserving water resources [17], and through photosystem II (PSII) and photosystem I (PSI) adaptations, which increase heat dissipation in the form of non-photochemical extinction, avoiding electron transfer obstruction and increased antioxidant enzyme activity, such as superoxide dismutase and catalase and ascorbate peroxidase, as important mechanisms to deal with oxidative stress [[18], [19], [20]]. Thus, this study has important parameters, such as those already mentioned, that can answer questions about the behavior of these plants when intercropped with annual crops and, above all, guide the selection of forest species for the system [21].

To understand the seasonal behavior of forest components, we studied a set of physiological and biochemical characteristics with a focus on the following hypotheses: (i) differential responses in physiological behavior among various forest species will be observed across seasons, influenced by the specific type of intercropping system diversification; (ii) The physiological behavior of tree species will be significantly impacted by the diversification of the intercropping system. Therefore, we estimated water relations, physiological performance, and stress indicators of two native species (*Anadenanthera macrocarpa* and *Dipteryx alata*), and two eucalyptus genotypes, Urograndis I-144 (*E. grandis* x *E. urophylla*) and Urocam VM01 (*E. urophylla* x *E. camaldulensis*), all three years old, cultivated in two intercropping systems (soybean and corn) and in the four seasons of the year (fall, winter, spring, and summer) to understand the relationship between the intercropping system and the physiological behavior of forest species arranged in the integration system.

## 2 Material and methods

### 2.1 Experimental design

**Fig. 1:**
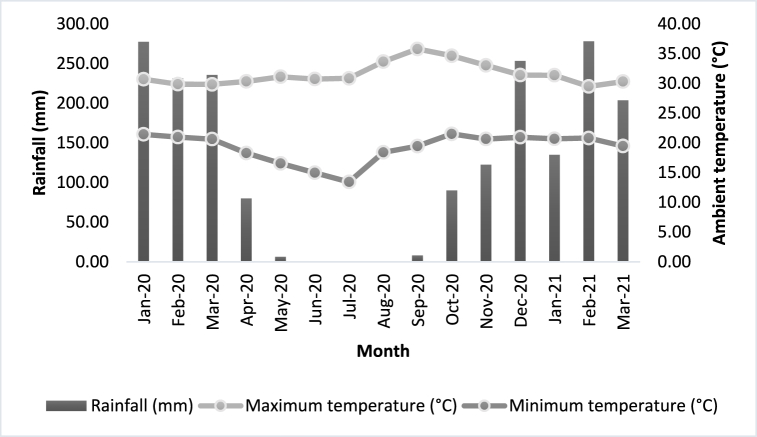
Average monthly temperatures and rainfall from January 2020 to March 2021 in the experimental area of the Instituto Federal de Ciência e Tecnologia Goiano, Iporá, Goiás, Brazil.

The field study was conducted from May 2020 to February 2021 in an experimental area of the Instituto Federal de Ciência e Tecnologia Goiano, in the city of Iporá, Goiás, Brazil (16°25′14.97″S, 51°9′22.15″W), elevated 600 m a.s.l. and with typical Cerrado vegetation. According to the Köppen-Geiger classification, the climate is tropical semi-humid (Aw), with two well-defined seasons during the year: wet summer, from December to March, and dry winter (June to September), with an average annual rainfall of 1402.1 mm [22]. The meteorological data was provided by the National Meteorological Institute [23] of the Iporá Meteorological Station (A028) and covers the experimental period (Fig. 1).

The soil is classified as Cambissolo. Table 1 describes the chemical characterization of the soil sampled before the experiment in the 0–20 cm depth layer.

**Table 1:** Chemical characteristics of the soil before the experiment. Abbreviations: CEC – Cation exchange capacity.

Soil chemical Properties												
pH	(g dm^−3^)	(mg dm^−3^)	--------(cmol dm^−3^)-------						V%		(%)	
^CaCl^_2_	OM	P	K	Ca	Mg	Al	H + Al	CEC	SB	Silt	Sand	Clay
4.50	10.14	1.2	0.22	0.88	0.28	0.2	4.12	5.50	26.4	25.0	29.8	44.8

CEC = Cation exchange capacity; OM = organic matter; SB = sum of the bases; V% = base saturation.

### 2.2 Forest species

The forest component studied consisted of two native species (*Anadenanthera macrocarpa* and *Dipteryx alata*) and two *Eucalyptus* genotypes, Urograndis I-144 (*E. grandis* x *E. urophylla*) and Urocam VM01 (*E. urophylla* x *E. camaldulensis*). The trees were planted in November 2018, with an east-west orientation, in single rows spaced 10 m apart. Spacing was defined according to the recommendations for each tree species: 3 m × 10 m (density of 333 plants ha^−1^) for the native species, and 2 m × 10 m (density of 500 plants ha^−1^) for the *Eucalyptus* genotypes, respectively.

**Fig. 2:**
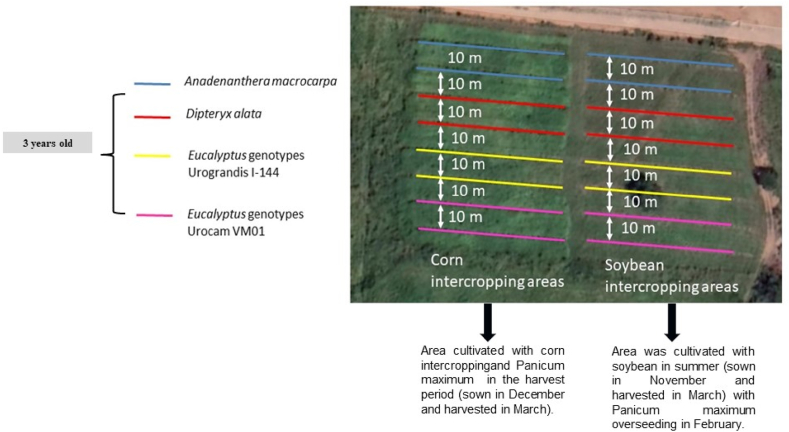
Description of the experimental area cultivated with soybean in summer (sown in November and harvested in March) with *Panicum maximum* overseeding in February and cultivated with corn intercropping and *Panicum maximum* in the harvest period (sown in December and harvested in March), where the forest component studied consisted of two native species (*Anadenanthera macrocarpa* and *Dipteryx alata*) and two Eucalyptus genotypes, Urograndis I-144 (*E. grandis* x *E. urophylla*) and Urocam VM01 (*E. urophylla* x *E. camaldulensis*).

The integrated system has a total area of 1 ha, divided into two experimental areas of 0.5 ha^-1^ each, where the four forest species were distributed in lines (orders: *A. macrocarpa*, *D. alata*, I-144 and VM01), and the lines spaced 10 m apart. One area was cultivated with soybean (cv. 'BRASMAX BÔNUS') cultivated with a plant spacing of 50 cm and a density of 180 thousand plants. They were planted in summer (sown in November and harvested in March) with *Panicum maximum* (cv. 'BRS Quênia') overseeding in February, using 6 kg ha^−1^. The other area was cultivated with corn intercropping (cv. 'P4285VYHR') cultivated with plant spacing of 50 cm and density of 63 thousand plants. And the same forage plant in the harvest period (sown in December and harvested in March). This intercropping was harvested for forage silage in the entire plot. Subsequently, both areas were grazed during winter (June to August) by dairy cows (Fig. 2).

### 2.3 Forest component evaluations

The physiological behavior of the native species, *Anadenanthera macrocarpa* and *Dipteryx alata*, and the *Eucalyptus* genotypes, Urograndis I-144 and Urocam VM01, were evaluated in soybean and corn intercropping areas in fall (May 2020) and winter (August 2020), which correspond to the dry season, and in spring (November 2020) and summer (February 2021), which correspond to the wet season.

#### 2.3.1 Water relations

Leaf water potential (_Ψw_) was measured in the morning (08:00–11:00) using a Scholander pressure chamber (3005-1412, Soilmoisture Equipment Corp., Goleta, CA, USA) [24].

#### 2.3.2 Chloroplast pigments

Chloroplast pigment concentrations were determined after extraction with dimethyl sulfoxide (DMSO) saturated with calcium carbonate (CaCO_3_) [25]. The concentrations of chlorophyll *a*, chlorophyll *b*, total chlorophyll and carotenoids were determined in a UV-VIS (Evolution 60S, Thermo Fisher Scientific Inc., MA, USA) at wavelengths of 665, 649, and 480 nm, respectively, calculated with the equations proposed by Wellburn [26] and with the results expressed in μg cm^2^ of leaf.

#### 2.3.3 Chlorophyll *a* fluorescence

Chlorophyll *a* fluorescence was measured using the FluorPen FP 100 portable fluorometer (Photon Systems Instruments; Drasov, Czech Republic). Young, fully expanded, non-detached leaves were previously adapted to the dark for 30 min for complete oxidation of the photosynthetic electron transport system. Minimum fluorescence (F_o_) was measured at 50 μs when all PSII reaction centers are open, being defined as the O step, followed by the J step (at 2 ms), the I step (at 30 ms), and maximum fluorescence (F_m_), when all PSII reaction centers are closed, known as the P step. The fluorescence variables were used to calculate several PSII bioenergetic indices: maximum primary photochemistry quantum yield (Phi_Po); quantum yield: the probability of a trapped exciton moving an electron down the electron transport chain after Qa- (Psio); electron transport quantum yield (PhiEO); quantum yield of energy dissipation in the form of heat (PhiDO); photosynthetic performance index (PiAbs); specific light energy absorption flux (ABSRC); maximum PSII capture rate (TRoRC); electron transport flux (in addition to Qa-) per reaction center with t = 0 (EToRC); specific energy dissipation flux at the chlorophyll level antenna (DioRC), following Strasser et al. [27].

#### 2.3.4 Gaseous exchange

Plant gas exchanges were evaluated to record photosynthetic (*A*, μmol CO_2_ m^−2^ s^−1^) and transpiration (*E*, mmol H_2_O m^−2^ s^−1^) rates, stomatal conductance *(g*_s,_ mol H_2_O m^−2^ s^−1^), and the ratio between internal and external CO_2_ concentration (*C*_i_*/C*_a_). Instantaneous water use efficiency (*WUE*) was obtained by the formula *WUE* = *A/E* (μmol CO_2_ mmol H_2_O^−1^), and carboxylation efficiency was calculated using the *A/C*_i_ ratio, respectively. These evaluations were conducted in a LI-6800 XT portable gas exchange meter (Li-Cor Inc., Nebraska, USA), between 08:00–11:00, at a block temperature of 25 °C and photosynthetic photon flux density of 1500 μmol m^−2^ s^−1^.

#### 2.3.5 Cell damage evaluation

Cell damage was evaluated by lipid peroxidation via malondialdehyde accumulation (MDA), as described by Cakmak and Horst [28], with some modifications. We added 1 mL of the reaction medium, consisting of 0.5 % (w/v) thiobarbituric acid (TBA) and 10 % (w/v) trichloroacetic acid (TCA), to test tubes containing 0.3 mL of the supernatant, which were then incubated at 90 °C for 20 min. The reactions were stopped in an ice bath and the samples centrifuged again at 10,000 g for 5 min. The samples were read in a UV-VIS spectrophotometer (Evolution 60S, Thermo Fisher Scientific Inc., MA, USA) at 440, 532, and 600 nm, and the content MDA was expressed as μmol MDA g^−1^ de MF.

#### 2.3.6 Activity of the antioxidant enzymes SOD, CAT, and APX

Superoxide dismutase (SOD), catalase (CAT), and ascorbate peroxidase (APX) activity was measured using approximately 0.3 g of ground leaf tissue macerated in liquid nitrogen and homogenized in potassium phosphate buffer solution (pH 6.8). The enzyme extract was centrifuged at 12,000 g for 15 min at 4 °C. The supernatant was used as a crude extract [29]. SOD activity (SOD - EC 1.15.1.1) was determined by measuring the enzyme ability to photochemically reduce nitrotetrazolium blue (NBT), according to Del Longo et al. [30] and determined at 560 nm in a spectrophotometer (Evolution 60S, Thermo Fisher Scientific Inc., MA, USA). An SOD unit was defined as the amount of enzyme needed to inhibit 50 % of NBT photoreduction [31]. CAT activity was determined by the rate of hydrogen peroxide (H_2_O_2_) degradation at 240 nm for 3 min at 25 °C [32], with some modifications. A molar extinction coefficient of 36 M^−1^ cm^−1^ [33] was used to calculate enzyme activity. APX activity was determined according to Nakano and Asada [34] and was measured by the rate of ascorbate oxidation at 290 nm for 1 min at 25 °C. A molar extinction coefficient of 2.8 mM^−1^ cm^−1^ [34] was used to calculate activity. All enzyme activities were expressed based on protein, whose concentration was determined by the method proposed by Bradford [35].

### 2.4 Statistical analysis

All statistical analyses were performed using R version 4.2.2 software [36]. The data was tested for normality by the Shapiro-Wilk test, using the *shapiro.test*(.) function. Outliers were identified and removed using the outlierTest(.) function of the ‘car’ package [37].

The data was subjected to analysis of variance using the aov(.) function, considering the effects of the system, forest species, season, and all of the interactions between them. When there was a significant effect (p < 0.05), the means were compared by the Tukey post-hoc test using the *emmeans(.)* function of the ‘emmeans’ package [38].

Subsequently, principal component analysis (PCA) was conducted for each forest species to assess the relationship between variables in each season. These analyses were conducted using the ‘FactoMineR’ [39] and ‘fatoextra’ [40] packages.

## 3 Results

The summary of the analysis of variance of the physiological and biochemical data of the indigenous forest species, *A. macrocarpa* and *D. alata*, and the *Eucalyptus* genotypes Urograndis I-144 and Urocam VM01 in soybean and corn intercropping areas evaluated in fall, winter, spring, and summer is shown in Supplementary Tables S1 and S2. Below, we present a detailed comparison of the results for each variable of interest, highlighting the differences between species in different intercropping conditions.

### 3.1 Water potential

**Fig. 3:**
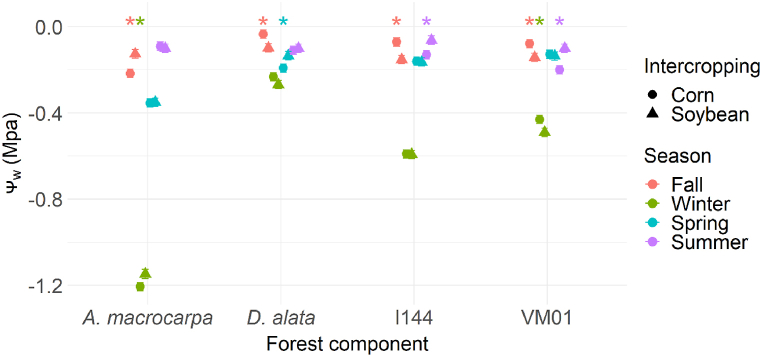
Morning leaf water potential (Ψw, MPa) of the species *Anadenanthera macrocarpa*, *Dipteryx alata*, and the *Eucalyptus* genotypes Urograndis I-144 and Urocam VM01 in soybean and corn intercropping areas evaluated in winter, fall, spring, and summer. Symbols and vertical bars represent means and standard errors, respectively (n = 5). * (p < 0.05) indicates species differences between intercrops in the particular season.

In the fall, a more negative water potential (_Ψw_) was observed for *A. macrocarpa* intercropped with corn and for *D. alata* and genotypes I-144 and VM01 intercropped with soybean (Fig. 3). *A. macrocarpa* intercropped with corn showed a more negative Ψ_w_ in winter (Fig. 3). On the other hand, genotype I-144 was significantly more negative when intercropped with corn in summer, while there was no difference between intercrops in spring (Fig. 3). Meanwhile, the VM01 genotype intercropped with soybean in winter and with corn in summer showed a more negative Ψ_w_ (Fig. 3).

### 3.2 Photosynthetic pigments

**Fig. 4:**
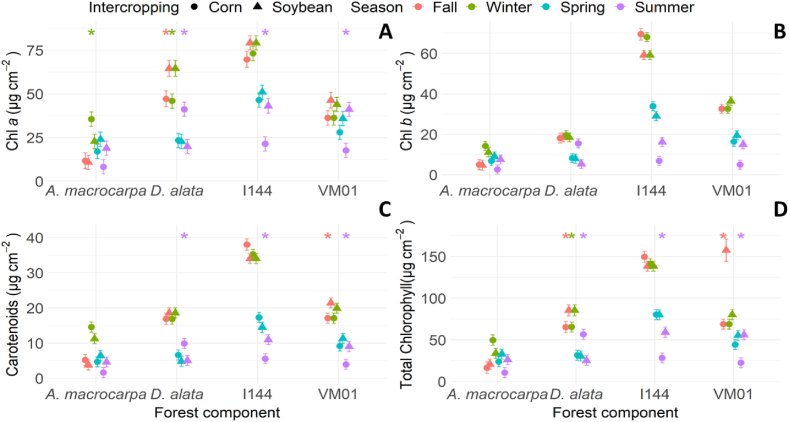
Chlorophyll *a* (Chl *a* = **A**), chlorophyll *b* (Chl *b* = **B**), carotenoids (**C**), and total chlorophyll (**D**) content of the species *Anadenanthera macrocarpa*, *Dipteryx alata*, and the *Eucalyptus* genotypes Urograndis I-144 and Urocam VM01 intercropped with soybean and corn evaluated in winter, fall, spring, and summer. Symbols and vertical bars represent means and standard errors, respectively (n = 5). * (p < 0.05) Indicates species differences between intercrops in the particular season.

Winter showed increased chlorophyll *a* concentration in *A. macrocarpa* intercropped with corn (Fig. 4A) compared to soybean intercropping. *D. alata* intercropped with soybean in winter and fall and intercropped with corn in summer also showed increased chlorophyll *a* concentration (Fig. 4A). Meanwhile, genotypes I-144 and VM01 presented increased chlorophyll *a* concentration in summer in the soybean intercropping area (Fig. 4A). No significant changes were observed for chlorophyll *b* (Fig. 4B). Carotenoid concentration was higher in *D. alata* intercropped with corn than with soybean in summer (Fig. 4C). Genotypes I-144 and VM01 intercropped with soybean showed significantly higher carotenoid concentrations than with corn in summer (Fig. 4C). There were no total chlorophyll concentration differences in *A. macrocarpa* (Fig. 4D). In *D. alata*, increased total chlorophyll values were observed in fall and winter in the soybean intercropping area and in summer in the corn intercropping area (Fig. 4D). In the *Eucalyptus* genotypes, increased total chlorophyll values were found in I-144 intercropped with soybean in summer, and in VM01 intercropped with soybean in fall and summer.

### 3.3 Chorophyll a fluorescence

**Fig. 5:**
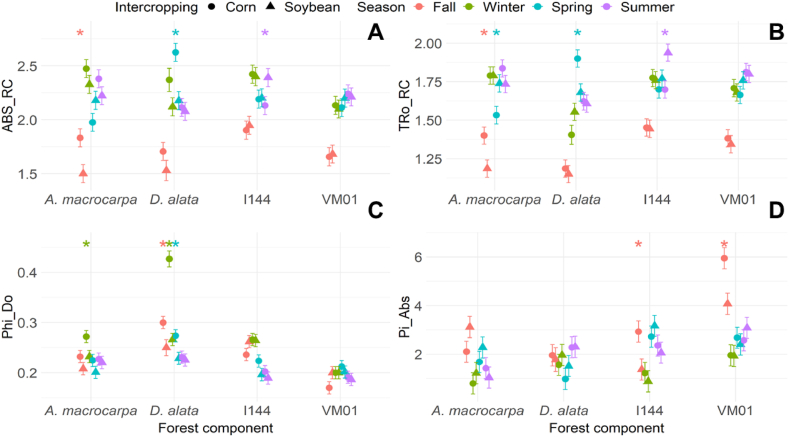
Specific light energy flux absorption (ABS_RC = **A**), maximum PSII capture rate (TRo_RC = **B**), quantum energy dissipation yield in the form of heat (Phi_Do = **C**), and photosynthetic performance index (Pi_Abs = **D**) of the species *Anadenanthera macrocarpa*, *Dipteryx alata*, and *Eucalyptus* genotypes Urograndis I-144 and Urocam VM01 intercropped with soybean and corn evaluated in winter, fall, spring, and summer. Symbols and vertical bars represent means and standard errors, respectively (n = 5). * (p < 0.05) Indicates species differences between intercrops in the particular season.

The native species *A. macrocarpa* and *D. alata* presented higher specific light energy absorption flux (ABS_RC) in corn intercropping in fall and spring (Fig. 5A). No significant changes were observed for ABS_RC in the VM01 genotype plants, regardless of intercropping and season (Fig. 5A). In contrast, genotype I144 showed higher ABS_RC values when intercropped with soybean in summer (Fig. 5A). The maximum PSII capture rate (TRo_RC) for *A. macrocarpa* was significantly higher in corn intercropping in fall (Fig. 5B) and in soybean intercropping in spring (Fig. 5B), with no difference between intercrops in winter and summer. In comparison, *D. alata* plants showed an increase in TRo_RC when intercropped with corn in spring (Fig. 5B). Genotype I-144 showed higher TRo_RC only when intercropped with soybean in summer (Fig. 5B), with no difference between intercrops in winter, fall, and spring (Fig. 5B). The VM01 genotype showed no differences between corn and soybean intercropping in any of the seasons (Fig. 5B). Likewise, no significant differences were observed in the energy dissipation quantum yield in the form of heat (Phi_Do, Fig. 5C) for the genotypes between intercrops or between seasons. An increased Phi_Do was observed in *A. macrocarpa* intercropped with corn in winter, while for *D. alata* it was higher in fall, winter, and spring when intercropped with corn (Fig. 5C). Higher photosynthetic performance indices (Pi_Abs) were only observed for genotypes I-144 and VM01 intercropped with corn in fall (Fig. 5D).

### 3.4 Gas exchange

**Fig. 6:**
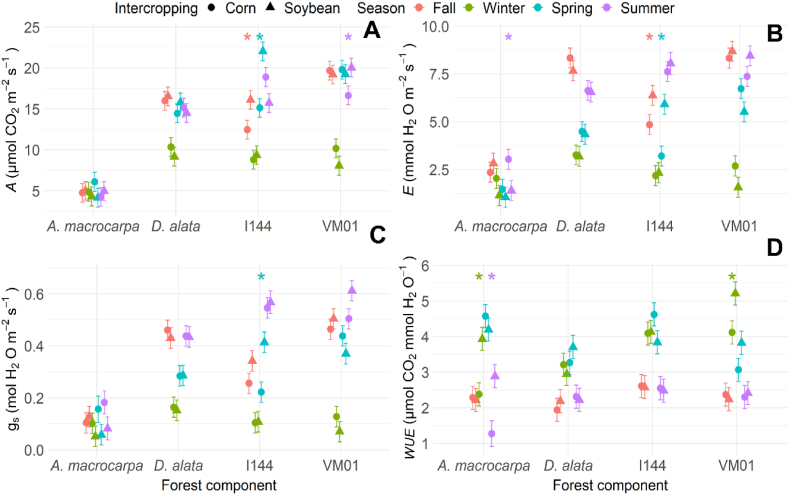
Photosynthetic rate *(A* = **A**), transpiration rate *(E* = **B**), stomatal conductance *(gs* = **C**), and instant efficiency in water use (*WUE* = **D**) of the species *Anadenanthera macrocarpa*, *Dipteryx alata*, and the *Eucalyptus* genotypes Urograndis I-144 and Urocam VM01 intercropped with soybean and corn evaluated in winter, fall, spring, and summer. Symbols and vertical bars represent means and standard errors, respectively (n = 5). * (p < 0.05) Indicates species differences between intercrops in the particular season.

Regarding the gaseous exchange variables, the photosynthetic rate (*A*) showed no differences between intercrops for *A. macrocarpa* and *D. alata* (Fig. 6A). A higher *A* was observed in I144 intercropped with soybean in fall and spring (Fig. 6A), while the VM01 genotype only showed elevated *A* levels when intercropped with soybean in summer (Fig. 6A). Improved *A* rates in genotype I-144 in spring were caused by increased *gs*, and consequently, increased leaf transpiration (*E*) rates (Fig. 6B and C). An increased *E* was also observed in *A. macrocarpa* intercropped with corn in summer (Fig. 6B), with no difference between intercrops or seasons in *D. alata* and genotype VM01 (Fig. 6B). As for the instantaneous *WUE*, the highest values were observed in *A. macrocarpa* intercropped with soybean in winter and summer (Fig. 6D) and in the VM01 genotype intercropped with soybean in winter (Fig. 6D).

### 3.5 Cell damage and enzyme activity

**Fig. 7:**
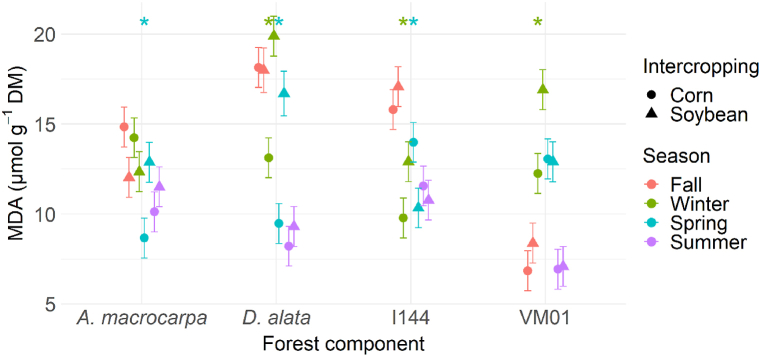
MDA concentration in the species *Anadenanthera macrocarpa*, *Dipteryx alata*, and the *Eucalyptus* genotypes Urograndis I-144 and Urocam VM01 intercropped with soybean and corn evaluated in winter, fall, spring, and summer. Symbols and vertical bars represent means and standard errors, respectively (n = 5). * (p < 0.05) Indicates species differences between intercrops in the particular season.

No changes in MDA content were observed in the native species and genotypes in fall and summer, regardless of the intercropping area (Fig. 7). MDA content was higher in *A. macrocarpa* only when intercropped with soybean in spring (Fig. 7). In *D. alata*, MDA showed higher accumulation in soybean intercropping in winter and spring (Fig. 7). In the I-144 genotype, MDA levels were higher for the intercrop with soybeans in winter and corn in spring (Fig. 7), while not differing in the other seasons. The VM01 genotype showed greater accumulation only when intercropped with soybean in winter (Fig. 7).

**Fig. 8:**
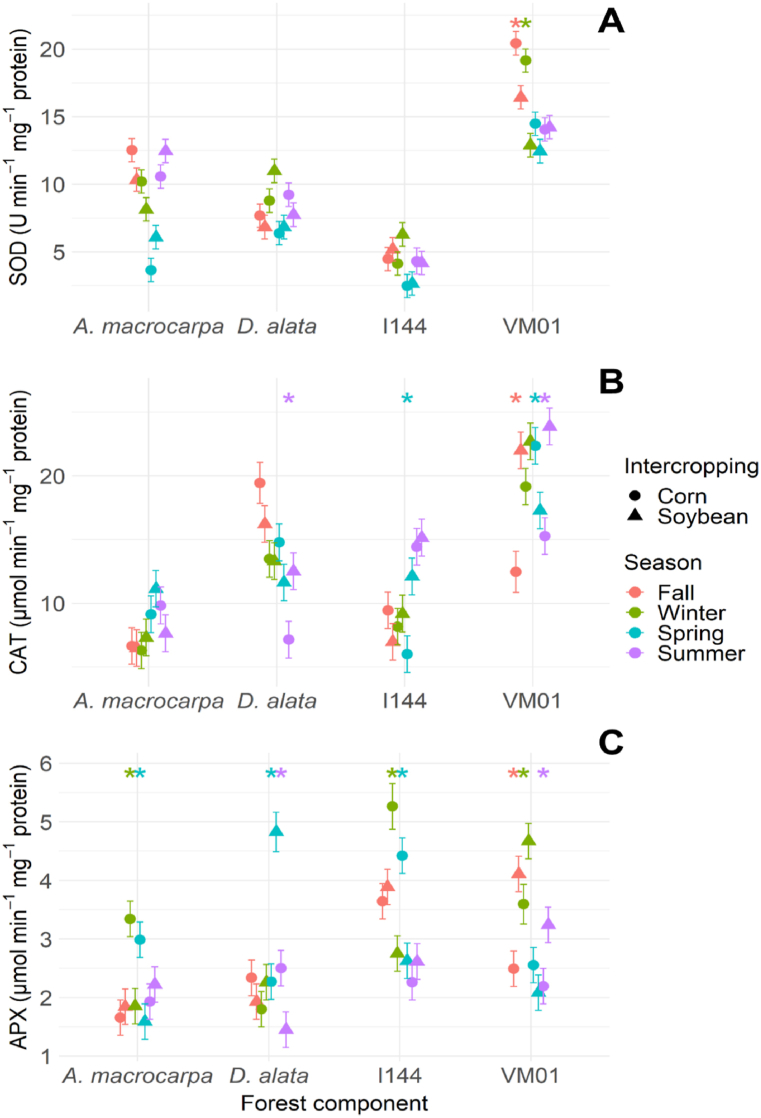
SOD (SOD = **A**), CAT (CAT = **B**), and APX (APX = **C**) activity in the species *Anadenanthera macrocarpa*, *Dipteryx alata*, and the *Eucalyptus* genotypes Urograndis I-144 and Urocam VM01 in soybean and corn intercropping areas evaluated in winter, fall, spring, and summer. Symbols and vertical bars represent means and standard errors, respectively (n = 5). * (p < 0.05) Indicates species differences between intercrops in the particular season.

Of the forest component antioxidant defense system enzymes (SOD, CAT, and APX), SOD showed the highest activity for the VM01 genotype in corn intercropping in winter and fall (Fig. 8A). Increased CAT activity was observed in *D. alata* intercropped with soybean in summer (Fig. 8B), in genotype I-144 intercropped with soybean in spring (Fig. 8B), and in genotype VM01 intercropped with soybean in fall and summer and with corn in spring (Fig. 8B). In contrast, APX activity was verified in all of the forest components (Fig. 8C). *A. macrocarpa* showed increased APX activity in corn intercropping in winter and spring (Fig. 8C), whereas *D. alata* showed increased activity in soybean and corn intercropping in spring and summer, respectively (Fig. 8C). Genotype I-144 showed higher APX activity in corn intercropping in winter and spring (Fig. 8C), and genotype VM01 in soybean intercropping in winter, fall, and summer (Fig. 8C).

### 3.6 Principal component analysis

**Fig. 9:**
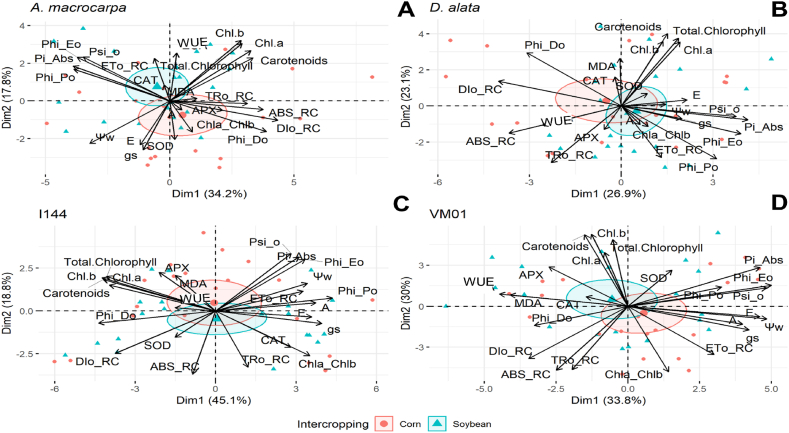
PCA representing the influence of physiological and biochemical variables in the native species *Anadenanthera macrocarpa* and *Dipteryx alata* and in *Eucalyptus* genotypes Urograndis I-144 and Urocam VM01 in soybean and corn intercropping areas in winter, fall, spring, and summer.

The soybean system increased the electron transport chain (Phi_Eo, Psi_o, Pi_Abs, and ETo_RC) and the *WUE* in *A. macrocarpa* (Fig. 9A). The corn system increased energy absorption per reaction center (ABS/RC) and the need for excess dissipation through non-photochemical dissipation pathways (Dio_RC, Phi_Do). In addition, there was increased antioxidant defense enzyme activity (SOD and APX) in the corn system. The results of the first two dimensions of the PCA for *D. alata*, I-144, and VM01 (Fig. 9B, C and 9D) showed no significant differences in the variables evaluated between soybean and corn intercrops.

## 4 Discussion

Understanding the physiological and biochemical behavior of any forest component in an integration system can be useful for predicting their competitive abilities throughout the seasons, and for choosing which management practice results in more balanced and productive intercropping. In this study, our hypothesis showed that intercropping system influences in the physiological and biochemical behavior variations of the four tree species in different seasons. The results indicate that growing native species such as *A. macrocarpa* in soybean intercropping increases their growth and developmental mechanisms, due to the absence of oxidative stress. *WUE* (Fig. 6D) was adjusted to reduce water loss and increased catalase activity (Fig. 8B), which is one of the main H_2_O_2_ elimination enzymes, with the potential to cause dismutation in up to six million H_2_O_2_ molecules per minute [41]. In addition, increased chlorophyll *a* fluorescence parameters, which are associated with better energy use for the electron transport chain (Pi_Abs, Psi_o, Phi_Po, Phi_Eo, and ETo_RC, Fig. 5, Fig. 9A) and high chloroplast pigment production (Fig. 4A–D), may be an adaptive strategy to protect tissues against radiation and oxidative stress and to increase photosynthetic efficiency [42]. This can be seen in the maintenance of the photosynthetic apparatus of *A. macrocarpa* in soybean intercropping, for example.

In contrast, when *A. macrocarpa* was intercropped with corn, although the species promotes high energy absorption per reaction center (Fig. 5, Fig. 9A), the increased SOD (Fig. 8A) and APX (Fig. 8C) activity occurred due to increased ROS levels, which induced oxidative stress (Fig. 7), and due to the greater need to dissipate excess energy through non-photochemical pathways (Dio_RC and Phi_Do; Fig. 5, Fig. 9A), resulting in reduced light use and CO_2_ assimilation [43,44]. Improved defense mechanisms (enzymatic and heat dissipation) can reduce the plant's growth capacity due to the negative effects on its metabolism [45]. This may occur in response to a more negative water potential (Fig. 3), which may be unfavorable for the species, especially during the dry season in winter, but may stabilize in the rainy season [46,47].

The opposite pattern was found for *D. alata*. PCA showed no significant differences between the systems (Fig. 9B). However, plants intercropped with corn maintained and stimulated a balance between light absorption by photosynthesis and excess energy dissipation, allowing leaf acclimatization [48], as evidenced by the increased Dio_RC and Phi_Do levels (Fig. 5C). Also, increased chloroplast pigments in fall (Fig. 4A–D), dissipation of the excess energy absorbed in winter (Fig. 5C), and greater electron transport in summer (Fig. 9B), even with a low electron absorption and capture flow (Fig. 5A), helped *D. alata* plants maintain their CO_2_ concentration capacity and maximize photosynthesis efficiency, even at low *WUE* and oxidative stress levels [49].

The Urograndis I-144 genotype showed no significant differences between intercrops (Fig. 9C), with a more negative Ψ_w_ in both of them (Fig. 3). Even with reduced _Ψw_, photosynthetic performance was maintained (Fig. 6A), which may be associated with stomatal adjustment *(gs*), in which the leaf turgor point is maintained even in periods of higher water demand [50]. The maintenance of relatively high *gs* values has been related to rapid growth in *Eucalyptus* under rapid and moderate water stress [51], but under severe and prolonged drought conditions, this characteristic will increase its vulnerability [52]. I-144 also showed high *WUE* in the drier seasons (winter and fall) (Fig. 6D), which helped it establish quickly at the start of the rainy season [53] and increase its chloroplast pigment production in the dry season (Fig. 4A–D). This behavior is an important adaptive strategy to protect leaf tissues against excess light and maximize photosynthetic efficiency, although not efficient, given the occurrence of oxidative stress (Fig. 7) [54]. In addition, increased energy dissipation as heat (Phi_Do, Fig. 5C) helped adjust CO_2_ fixation rates in the dry seasons, indicating that the genotype can grow partially (although not optimally) in water-restricted environments [55]. However, changes in PSII action will result in reduced carbon assimilation in longer or more intense drought events, which can be expected with increased climate change events [56,57]. This will affect the photosynthetic process and, consequently, the productivity and survival of this genotype [58].

As for the Urocam VM01 genotype, PCA (Fig. 9D) showed similar behavior to I-144 in terms of physiological and biochemical responses, which were not influenced by the intercropping system. This genotype was developed for cultivation in the Cerrado, being tolerant to abiotic stress conditions, such as water deficit [59]. Avoidance or tolerance to low water availability was observed in winter and fall (Fig. 3), in addition to mobilization of physiological pathways to avoid damage to photosynthetic processes in order to survive and await conditions more conducive to optimal development [60]. VM01 tried to reduce the effects of water deficit by minimizing water loss and/or optimizing its acquisition through the roots, thus leading to increased *WUE* (Fig. 6D) [25]. VM01 exposure to water stress also resulted in oxidative damage in winter (Fig. 7), requiring plant defenses to tolerate this condition, especially through SOD, CAT, and APX activation (Fig. 8A–C) [61]. Even under oxidative stress, there was no damage to the photosynthetic apparatus (Fig. 5A–D), demonstrating this genotype's ability to maintain an even when subjected to drought, which indicates its resistance to drought conditions [43]. The good performance of the photosynthetic apparatus is mainly due to the increased chloroplast pigments (Fig. 4A–D) that maintained efficient PSII functioning and the full use of the energy captured [62]. As for resilience, the genotype recovered and developed after the drought period at the start of the rainy season. Wood production management is one of the main objectives of the forest component in integration systems. Despite their robustness and adaptability to the drought conditions in the Cerrado, the native species *A. macrocarpa* in corn intercropping and *D. alata* in both intercrops have limitations that are mainly correlated with key processes, such as *WUE* adjustments, photosynthetic performance, and antioxidant system action, factors that limit their growth in integration systems in the drier seasons. In contrast, the eucalyptus genotypes showed more aggressive behavior, suggesting that the exotic species tend to present mechanisms in the intercropping system that adjust their growth, even in drought conditions [63]. This is due to their robustness, genetically improved properties, and their physiological adaptations that can improve resource acquisition in integration systems [64,65]. However, in the long term, especially considering climate changes, this could reduce their performance in integration systems, as they easily deplete soil water sources, harming the system as a whole [66].

## 5 Conclusions

These results describe potential hotspots of vulnerability for the native species *A. macrocarpa* in a corn system and for *D. alata* in both soybean and corn systems. This is reflected by greater investment in activations of pathways to dissipate excess energy and oxidative damage and activity of defense enzymes. Genotype I-144 presented changes in the functioning of PSII that under prolonged drought conditions could compromise the photosynthetic process, productivity and survival of the genotype. Consequently, both the native species and the I-144 genotype should receive special attention in the context of adaptations to anticipated climate change. In this sense, the VM01 genotype is more adaptable to the system, as it uses captured energy, minimizing or optimizing water loss and demonstrating greater resilience.

## Funding

This work was supported by the 10.13039/501100003593National Council for Scientific and Technological Development (10.13039/501100003593CNPq) (grant nos. 400901/2019-6 and 160186/2020-0).

## Data availability

Data will be made available on request.

## CRediT authorship contribution statement

**Érica Letícia Gomes Costa:** Writing – review & editing, Writing – original draft, Validation, Methodology, Investigation, Formal analysis, Data curation, Conceptualization. **Thales Caetano de Oliveira:** Writing – review & editing, Writing – original draft, Visualization, Validation, Methodology, Investigation, Formal analysis, Data curation, Conceptualization. **Alex Rodrigues Gomes:** Writing – review & editing, Methodology, Investigation, Conceptualization. **Carlos Henrique Pereira Bento:** Writing – review & editing, Methodology, Investigation, Conceptualization. **Fabia Barbosa da Silva:** Writing – review & editing, Writing – original draft, Methodology, Investigation, Formal analysis, Data curation. **Estenio Moreira Alves:** Writing – review & editing, Methodology, Investigation, Conceptualization. **Tiago do Prado Paim:** Writing – review & editing, Writing – original draft, Validation, Software, Methodology, Conceptualization. **Fabiano Guimarães Silva:** Writing – review & editing, Resources, Project administration, Funding acquisition, Conceptualization.

## Declaration of competing interest

The authors declare the following financial interests/personal relationships which may be considered as potential competing interests:Fabiano Guimaraes Silva reports financial support was provided by National Council for Scientific and Technological Development. If there are other authors, they declare that they have no known competing financial interests or personal relationships that could have appeared to influence the work reported in this paper.
