# Supplementary material for: Using seasonal physiological and biochemical responses to select forest components adapted to soybean and corn intercropping
Source: Heliyon. 2024 Jul 24;10(16):e34674. doi: 10.1016/j.heliyon.2024.e34674 (PMC11367055; doi:10.1016/j.heliyon.2024.e34674)
Supplement: Multimedia component 1 [file mmc1.docx]

**Table S1.** Summary of the analysis of variance of water potential (_Ψw_) physiological data. Chloroplast pigments: Chlorophyll *a* (Chl *a*), chlorophyll *b* (Chl *b*), carotenoids (Car), and total chlorophyll content (TChl); chlorophyll *a* fluorescence: maximum primary photochemistry quantum yield (in t = 0) (Phi­_Po); quantum yield: the probability (t = 0) of a trapped exciton moving an electron down the electron transport chain after Qa- (Psio); electron transport quantum yield (PhiE_O_); quantum yield of energy dissipation in the form of heat (PhiD_O_); photosynthetic performance index (PiAbs); specific light energy absorption flux (ABSRC); maximum FSII capture rate (_TRoRC_); electron transport flux (in addition to Qa-) per reaction center with t = 0 (EToRC); specific energy dissipation flux at the chlorophyll level antenna (_DioRC_). Gaseous exchanges: Photosynthetic rate (*A*); transpiration rate (*E*); stomatal conductance (*gs*), and instantaneous WUE of the forest species *Anadenanthera macrocarpa*, *Dipteryx alata*, and the eucalyptus genotypes Urograndis I 144 and Urocam VM01 in soybean and corn intercropping areas evaluated in winter, fall, spring, and summer.

| **FV** | **GL** | Ψ_w_ | ***A*** | **Chl*a*** | **Chl*b*** | **Car** | **TChl** | **PhiPo** | **Psio** | **PhiEo** | **PhiDo** | **PiAbs** | **ABSRC** | **TRoRC** | **EToRC** | **DIoRC** | ***A*** | ***E*** | ***gs*** | ***EUA*** |
| --- | --- | --- | --- | --- | --- | --- | --- | --- | --- | --- | --- | --- | --- | --- | --- | --- | --- | --- | --- | --- |
| **Tree** |  | * | NS | * | NS | NS | NS | * | NS | NS | * | NS | NS | NS | NS | * | NS | NS | NS | *** |
| **Tree Distance** |  | *** | ** | *** | NS | *** | *** | *** | *** | *** | *** | NS | * | *** | * | *** | ** | NS | NS | *** |
| **Growth Stages** |  | *** | NS | *** | NS | *** | *** | * | NS | NS | * | NS | *** | *** | NS | *** | NS | NS | NS | *** |
| **Tree:Tree Distance** |  | * | NS | ** | NS | NS | * | *** | NS | *** | *** | NS | NS | NS | ** | *** | NS | NS | NS | * |
| **Tree:Growth Stages** |  | * | NS | * | NS | NS | NS | NS | NS | NS | NS | NS | * | ** | * | NS | NS | NS | NS | ** |
| **Tree Distance:Growth Stages** | 6 | *** | *** | *** | NS | *** | *** | *** | *** | * | *** | *** | *** | *** | NS | *** | *** | *** | *** | *** |
| **Tree:Tree Distance:Growth Stages** | 7 | *** | ** | *** | ** | ** | *** | ** | NS | * | *** | NS | * | ** | ** | *** | ** | NS | NS | * |

int: intercropping; treat: treatment; sea: season; GL: degree of freedom; *: p < 0.05; **: p < 0.01; ***: p < 0.001; NS: not significant.

**Table S2.** Summary of the analysis of variance of data on cell damage (MDA) and enzymatic activity of the antioxidant defense enzymes SOD, CAT, and APX in the forest species *Anadenanthera macrocarpa*, *Dipteryx alata*, and the eucalyptus genotypes Urograndis I 144, and Urocam VM01 in soybean and corn intercropping areas evaluated in winter, fall, spring, and summer.

| **FV** | **GL** | **MDA** | **SOD** | **CAT** | **APX** |
| --- | --- | --- | --- | --- | --- |
| **Tree** |  | NS | NS | NS | *** |
| **Tree Distance** |  | * | *** | *** | *** |
| **Growth Stages** |  | *** | *** | NS | *** |
| **Tree:Tree Distance** |  | ** | *** | NS | *** |
| **Tree:Growth Stages** |  | ** | ** | NS | ** |
| **Tree Distance:Growth Stages** |  | *** | *** | *** | *** |
| **Tree:Tree Distance:Growth Stages** |  | *** | ** | *** | *** |

int: intercropping; treat: treatment; sea: season; GL: degree of freedom; *: p < 0.05; **: p < 0.01; ***: p < 0.001; NS: not significant.
